# Direct insight into the structure-property relation of interfaces from constrained crystal structure prediction

**DOI:** 10.1038/s41467-020-20855-0

**Published:** 2021-02-05

**Authors:** Lin Sun, Miguel A. L. Marques, Silvana Botti

**Affiliations:** 1 Institut für Festkörpertheorie und -optik, Friedrich-Schiller-Universität Jena, Jena, Germany; 2 Institut für Physik, Martin-Luther-Universität Halle-Wittenberg, Halle, Germany; 3 European Theoretical Spectroscopy Facility,

**Keywords:** Materials chemistry, Computational methods, Electronic structure, Electronic properties and materials

## Abstract

A major issue that prevents a full understanding of heterogeneous materials is the lack of systematic first-principles methods to consistently predict energetics and electronic properties of reconstructed interfaces. In this work we address this problem with an efficient and accurate computational scheme. We extend the minima-hopping method implementing constraints crafted for two-dimensional atomic relaxation and enabling variations of the atomic density close to the interface. A combination of density-functional and accurate density-functional tight-binding calculations supply energy and forces to structure prediction. We demonstrate the power of this method by applying it to extract structure-property relations for a large and varied family of symmetric and asymmetric tilt boundaries in polycrystalline silicon. We find a rich polymorphism in the interface reconstructions, with recurring bonding patterns that we classify in increasing energetic order. Finally, a clear relation between bonding patterns and electrically active grain boundary states is unveiled and discussed.

## Abstract (web summary)

The prediction of atomic structure at interfaces is a challenging problem in material science. Here, the authors demonstrate a new algorithm for global structure prediction of interface reconstructions by successfully identifying atomic arrangements in symmetric and asymmetric tilt boundaries in polycrystalline silicon.

## Introduction

The prediction of microscopic atomic structures of interfaces, and the determination of their effects on the electronic properties of a multicomponent material is a challenging open problem of materials science. A solution to this scientific question is of utmost importance to understand, e.g., how to control the overall electronic response of a functional material. An ubiquitous example of internal interfaces are grain boundaries (GBs), i.e. two-dimensional defects that separate crystalline domains with different orientation in a polycrystalline sample^1^. GBs are present even in the purest samples and can affect dramatically structural, electronic, transport, and optical properties of semiconducting crystals used in microelectronic devices or solar cells^2–11^. In the latter devices, in particular, the effect of GBs is generally considered to be detrimental to charge transport, as deep defect states at internal interfaces can act as recombination centers for excited electrons and holes. However, the introduced disorder has a beneficial effect on light absorption efficiency, as it allows to break the *k*-point selection rule for optical transitions^12^. A deep understanding of low-enthalpy structural reconstructions at GBs would provide the missing insight on how GBs limit energy conversion efficiency, disclosing new routes to enhance both light absorption and charge mobility in photovoltaic devices.

The unresolved incongruities in the large body of experimental and theoretical work available on GBs reinforce a number of fundamental questions concerning the relation between the symmetry of the GB and the measured charge carrier mobility or mechanical strength^13,14^. Due to the structural complexity of interfaces, the investigation of the precise link between material performance and interface reconstructions remains a challenging task for experimental techniques and computer simulations. Thanks to recent advances in nanocrystalline engineering, it is now possible to incorporate specific interfaces in a material^15,16^. Direct experimental observations of interfacial geometries by high-resolution transmission electron microscopy are extremely difficult^17^, but, when they are successful, they generally show that GB atomic structures can bear little resemblance to intuitively guessed atomic configurations^18^. In many cases, experimental images do not provide sufficient information about the atomic level structure, so that, without theoretical support, it remains debated whether certain GBs are ordered, partially ordered, amorphous, or liquid^19^. Unfortunately, and despite the large amount of theoretical work in literature^20–33^, a comprehensive understanding of how GB reconstructions determine the electronic properties of a realistic polycrystal is still missing.

The main difficulty is that experimental data are incomplete and simulations do not always sample the configuration space in an adequate manner. The introduction of genetic algorithms for the prediction of interface reconstructions^28,29^ represented a significant advance. Zhang et al.^28^ combined a genetic algorithm with classical molecular dynamics and tight-binding to study silicon GBs. The use of quantum tight-binding methods led to substantial improvement of the reliability of calculations, as classical potentials are often inaccurate, and sometimes even qualitatively wrong^32,34^, when it comes to sample the complex multi-dimensional potential energy surface. Chua et al.^29^ developed a genetic algorithm to study both stoichiometric and non-stoichiometric GBs of SrTiO_3_^29^. Due to the high computational cost, classical interatomic potentials were preferred to carry out this work^29^. More recently, algorithms and codes developed for structural prediction of bulk materials were adapted to tackle the challenging problem of predicting interface reconstructions. Zhu et al.^19^ extended USPEX^35,36^, a well-established and robust crystal structure prediction code, to the structural prediction of interfaces. In their seminal work^19^, they combined evolutionary search and classical molecular dynamics, boosted by machine learning post-processing analysis, to uncover an unexpected rich polymorphism of Cu tilt GBs^19,37,38^. Genetic algorithms together with classical potentials were similarly successfully employed to investigate GBs in SrTiO_3_^29,39^ and symmetric tilt and twist GBs in elementary body-centered cubic metals^40^. The reduced computational cost provided by the use of classical potentials enabled the reconstruction and classification a large set of boundaries, accounting for variable atomic densities at the interface. Similar low-energy reconstruction patterns were also determined in other metallic GBs, combining structural relaxation using classical potentials with Monte Carlo probabilities of atom addition or removal^41^.

The stabilizing effect of a reduced atomic density at the interface had already been discussed in early studies on twist GBs in ionic oxides by Tasker and Duffy^42^. Specifically concerning GBs in silicon, this important issue was stressed by Von Alfthan et al.^43–45^ that, using classical molecular dynamics, revealed low-energy configurations with a higher degree of structural diversity by varying the number of atoms in the interface region. GB energies were then calculated a posteriori using density functional theory (DFT). In view of those findings, previous simulations of GBs in silicon that did not consider variable atomic densities at the interface were disputed.

Gao et al.^46^ have recently adapted the particle swarm method for crystal structure prediction to determine GB reconstructions, presenting applications for graphene and rutile TiO_2_^46^. Bonding constraints were imposed to generate better starting structures and DFT tight-binding was used to obtain energies and forces. As standard Slater–Koster parameters provided by the DFTB+ distributions^47^ were adopted, the less accurate tight-binding calculations were complemented by DFT reoptimization of the lowest-energy structures. Addition and removal of atoms in the interface region during structural prediction were not considered.

Random structure searching for interfaces was proposed by Schusteritsch and Pickard^48^. This method relies on the repeated generation of random atomic positions in the vicinity of an interface, respecting efficient constraints, followed by relaxations using DFT energies and forces. Results for GBs in graphene and SrTiO_3_ were discussed in both stoichiometric and non-stoichiometric conditions. The computational cost involved, especially for the three-dimensional system, was considerable, and in fact only one tilt GB—Σ3(111)—was discussed in this work. Moreover, to ease the computational burden, structural search was performed using coarse parameters and soft pseudopotentials, and only the relevant structures were refined by final accurate calculations.

All in all, a comparison of the different technical choices underlying these multiple studies brings to light complementary insight on the mechanisms of interface reconstruction. We can learn important lessons from the analysis of previous literature. First of all, varying the atomic density at a grain boundary is a key step: in fact, some thermodynamic states, that are accessed in real systems by diffusion processes, can be simulated in a supercell model only by removing or adding atoms. Significant deviations from DFT results have been demonstrated for calculations using classical potentials for silicon^49^. It is also known that force-fields are not capable to stabilize the lowest energy point defect in silicon^50^. A recent comparison with DFT results for a large dataset also shows that forces calculated with Stillinger–Weber or Tersoff force-fields are of poor quality, both in magnitude and in direction^51^, pointing to the need of a quantum mechanical description of energies and forces. However, DFT calculations remain too costly and different strategies for computational efficiency need to be followed to ensure the accuracy of quantum ab initio calculations of energies and forces, as well as the possibility to access large families of GBs. In this work, we use tight-binding parameters optimized to reproduce a large dataset of DFT calculations that guarantee a good sampling of the potential energy surface and yield DFT-quality energy and forces^52^.

The development of an accurate and unbiased first-principle procedure for the extraction of structure–property relations of families of interfaces is the subject of this Article. We propose here an efficient approach for the prediction of low-energy reconstructions of interfaces, that retains and combines strong points of previous approaches and tries to go beyond still existing limitations. Our algorithm is based on the minima-hopping method^53,54^ (MHM) for global crystal structure prediction, that we modified to direct the search away from the global minimum. In the same spirit, recent developments have allowed to use the MHM for structural prediction of surfaces^55^, low-density structures^56^ and two-dimensional materials^57–59^. To enforce the search of GB geometries with the lowest surface energies, we introduce appropriate geometrical constraints for bulk-like regions. Moreover, we permit variable atomic densities in the interface region, to account for atomic diffusion processes relevant for the minimization of interface energies and strain. A clear advantage of the constrained MHM with respect to constrained evolutionary algorithms, is that physical constraints have a more intuitive form and are immediate to implement.

To verify the performance of the variant MHM for interfaces, we apply it to study a set of low-energy tilt GBs in silicon. The focus on this family of GBs is motivated by the large amount of data available in the literature for polycrystalline silicon, which makes this system ideal for a thorough validation of the proposed approach. Our objective is, on one hand, to identify GB phases at a lower energy than those already reported and/or in better agreement with available experimental data. On the other hand, we want to collect comprehensive information on recurrent patterns in low-energy interface reconstructions for silicon, and use these patterns to build a systematic classification of Si GB.

## Results and discussion

### Low-energy GBs in silicon

Several types of GBs were known to be present in polycrystalline silicon^31,32,60,61^. The majority has relatively high coherence, which is described by a low-Σ value of the associated coincidence site lattice. In this Article we use the so-called “axis-angle” notation to specify the grain boundary lattice misorientation, i.e. we define a crystallographic surface (hkl) (or two if the surface is not common to the two crystal grains), and a rotation angle *θ* about the axis *n*. Twist boundaries and tilt boundaries are subgroups of GBs with special orientations of the axis *n*: for twist GBs [hkl] and *n* are parallel, while tilt GBs have the *n* axis in the surface plane.

Twist GBs are usually easier to build and relax, as the rotation axis is perpendicular to the cut plane and there is no need to remove or add material at the interface after the rotation. We decided therefore to focus on the case of tilt boundaries, which have rotation axis lying in the cut plane. In these systems, strain and under- or over-coordination at interfaces can already play an important role, while the level of complexity (i.e., the supercell size) remains controlled.

The most frequently encountered GBs are the energetically favored coincidence site lattices Σ3, Σ9, and Σ27, with Σ3 GBs accounting for around 50% of the internal interfaces in multi-crystalline samples^13,61–63^. Note that the Σ3(111) GB is mirror symmetric and can be obtained either as a tilt boundary, by performing a rotation of 70.5° around the [110] axis, or as a twist boundary, by a rotation 60° around the [111] axis. Σ3(111) is the most stable GB in polycrystalline silicon. The reason is easy to understand: due to its high symmetry, it preserves the bond lengths and angles of the bulk crystal.

**Fig. 1:**
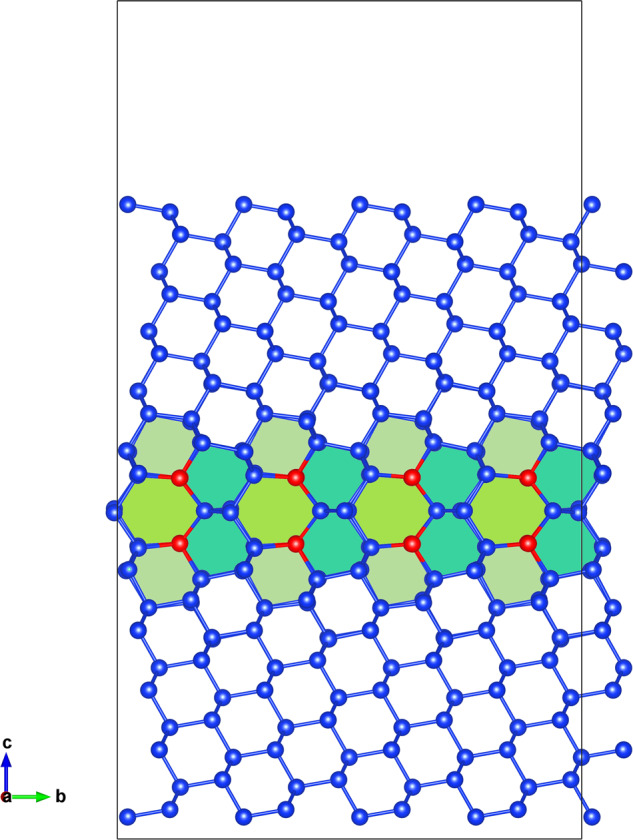
The [110] projection of the reconstructed Σ11(113) interface. This supercell contains 336 atoms. Single-atomic columns aligned along the [110] axis, i.e. perpendicular to the view plane, are highlighted by red atoms.

In the following we consider coincidence site lattices with Σ ≤ 29. These structures display a significant variety of starting interface configurations and have often been detected in experimental samples^13,31,60,61^. A summary of the structures under investigation, with information on their tilt axis and angle, is given in Table 1. The total number of silicon atoms in the considered unit cells ranges from 96 to more than 400. In Supplementary Table 2 we provide more information on all supercells used in this study. An example of supercell that models a single grain boundary of Σ11(113) is shown in Fig. 1.

**Table 1:** Summary of the structures studied in this work: GB labels, tilt axis, misorientation angle *θ*, and minimum GB energy *γ* in J/m^2^) after interface reconstruction.

Label	Tilt axis	*θ*	*γ*
Σ13a(051)	[100]	22.6°	0.672
Σ19a(331)	[110]	26.5°	0.339
Σ17a(041)	[100]	28.1°	0.601
Σ27a(552)	[110]	31.6°	0.381
Σ5(031)	[100]	36.9°	0.358
Σ9(221)	[110]	38.9°	0.202
Σ29a(052)	[100]	43.6°	0.536
Σ11(332)	[110]	50.5°	0.392
Σ5(021)	[100]	53.1°	0.393
Σ13a(032)	[100]	67.4°	0.614
Σ3(111)	[110]	70.5°	0.013
Σ25a(043)	[100]	73.7°	0.646
Σ17b(334)	[110]	86.6°	0.462
Σ17b(223)	[110]	93.4°	0.440
Σ3(112)	[110]	109.5°	0.386
Σ11(113)	[110]	129.5°	0.402
Σ9(114)	[110]	141.1°	0.378
Σ27a(115)	[110]	148.4°	0.623
Σ19a(116)	[110]	153.5°	0.616
Σ3(001 × 221)	[110]	70.5°	0.497
Σ9(111 × 115)	[110]	38.9°	0.433

### Constrained minima-hopping for interfaces

Our structural optimization method is based on the MHM^53,54^, an efficient structural prediction algorithm designed to determine the low-energy crystal structures of a system given solely its chemical composition. In this approach, the energy surface is explored by performing consecutive short molecular dynamics escape steps followed by local geometry relaxations, taking into account both atomic and cell variables. The predictive power of this approach was already demonstrated in a wide set of applications, ranging from bulk crystals^64^ to low-density structures^56^, crystals with defects^65^, quasi-two-dimensional materials^57^, etc.

The number of atoms per unit cell for the simulation of interfaces is necessarily much larger than the typical number of atoms in the unit cell of the underlying perfect crystals. In view of that, energies, forces, and strains are more conveniently computed using density-functional tight-binding (DFTB)^47,52,66^. For a fully reliable interpretation of the results, the final structures are always refined using DFT.

To adapt the MHM to the calculation of lowest-energy interface reconstructions, we implemented efficient force constraints that act on atoms in the bulk region and allow for variable atomic densities close to the interface. To this end, we separate the atoms into two sets, namely those belonging to the bulk regions (displaying the typical tetrahedral coordination of diamond silicon) and the ones making part of the interface. While the coordinates of interface atoms are fully relaxed applying the minima-hopping algorithm, the bulk layers are constrained to move as a rigid body, i.e. bond lengths and angles are fixed, but it is still possible to minimize the strain on the atoms belonging to the interface region during their reconstruction. These constrains are applied in both the molecular dynamics and the relaxation steps. Moreover, atoms are systematically added or removed when the initial interface configurations are prepared, to vary the atomic density of the interface region. We tested against convergence the thickness of bulk and interface regions and we found, as a rule of thumb, that the number of interface atoms should be about half the total number of atoms in the supercell.

The task of the MHM runs is to find the arrangements of interface atoms that minimize the interface energy, i.e. the difference of total energy per unit area between the supercell with a GB and a supercell with the same number of atoms of crystalline silicon. We make use of supercells with and without vacuum layers to extract the interface energy of a single reconstructed GB.

More information on the calculations, together with a detailed description of the constrained method for structural prediction of interfaces, and specifically of the procedure that we adopt for the evaluation of the interface energy, is presented in the “Methods” section.

### Classification of reconstruction patterns

Our calculations indicate that almost all considered GBs present a strong reconstruction of atomic bonding at the interface, that completely modifies the initial welding of tilted crystalline grains. Such reconstructions cannot be obtained by performing standard structural optimizations: a local relaxation would in fact only lead to the closest local minimum, totally ignoring the complexity of the highly dimensional potential energy surface. An exception is the very symmetric Σ3(111) GB, where all silicon atoms are already conveniently bonded from the start. Not surprisingly, the reconstructed geometries always tend to eliminate dangling bonds, and to bring Si–Si bond lengths and bond angles as close as possible to their value in bulk diamond silicon.

**Fig. 2:**
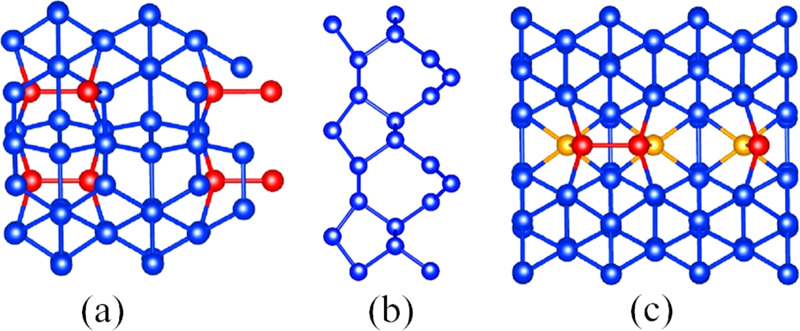
Recurring reconstruction patterns. **a** A [11$\bar{1}$] view of the ground state of the Σ3(112) GB, showing reconstructed single atomic columns (red atoms), aligned along the [110] rotation axis. **b** Atomic spirals along the [100] rotation axis in a Σ5(021) GB. **c** View of a [111] plane of a low-energy reconstruction of the Σ3(112) GB with five-coordinated silicon atoms (orange atoms) and single atomic columns (red atoms).

To label and classify the reconstructed GBs we extend the set of structural units and the notation proposed by Papon and Petit^67^. If we draw [110] projections of the stick-and-ball models of the interface, as in Fig. 1, we can see that *sp*^3^ hybridized atoms form cyclic units, whose composing rings can be labeled by the symbols ${\,}^{k}{n}_{ai}^{j}$. The notation is easy to interpret: *n* is the number of edges of the ring, *i* is the number of single atomic columns (see panel a of Fig. 2), *a* indicates either a boat shape (*a* = “b”) or a nearly flat shape (*a* = “f”) in the lateral view of the bonding pattern, and *j* is the number of double lines (if *j* = 2, this index is omitted). A double line, that sometimes is really indicated as a double line in the stick-and-ball model, is the bonding of an atom of the basic (110) plane with two others atoms situated in the upper and lower planes. It turned out that our simulations yielded a few extra bonding patterns that were absent in the classification of ref. ^67^. These new units are related, specifically, to the existence of silicon atoms with fivefold coordination (see panel c of Fig. 2). In view of that, we added the superscript *k* = 5 to indicate the presence of fivefold-coordinated atoms in the ring. We never found, in most stable reconstructed GBs, silicons atoms with <4 nearest neighbors (i.e., with dangling bonds), or with more than 5 nearest neighbors. These units appear only in very high-energy configurations that are quickly dismissed by our structural prediction procedure.

We observe that in many cases the lowest-energy reconstruction is attained only after adding (or removing) atoms to (from) the interface region. We remark that we tested systematically all possible modifications of the atomic density, until reaching a configuration equivalent to the starting one, differing by the simple addition of a full atomic plane.

The most common unit found in the lowest-energy reconstruction patterns is, not surprisingly, the standard bulk six-membered ring. This is followed by five-membered rings and 6_b_ and 6_b1_ six-membered rings. Also 6_1_ and 7_1_ units appear with a high frequency. Note that 6_b1_, 6_1_, and 7_1_ rings include one single atomic column, making this particular bonding scheme rather common in our reconstructed interfaces. A detailed summary of all relevant reconstruction patterns, with information regarding the frequency of each unit ring, can be found in Supplementary Fig. 3 and Supplementary Tables 3–5.

**Fig. 3:**
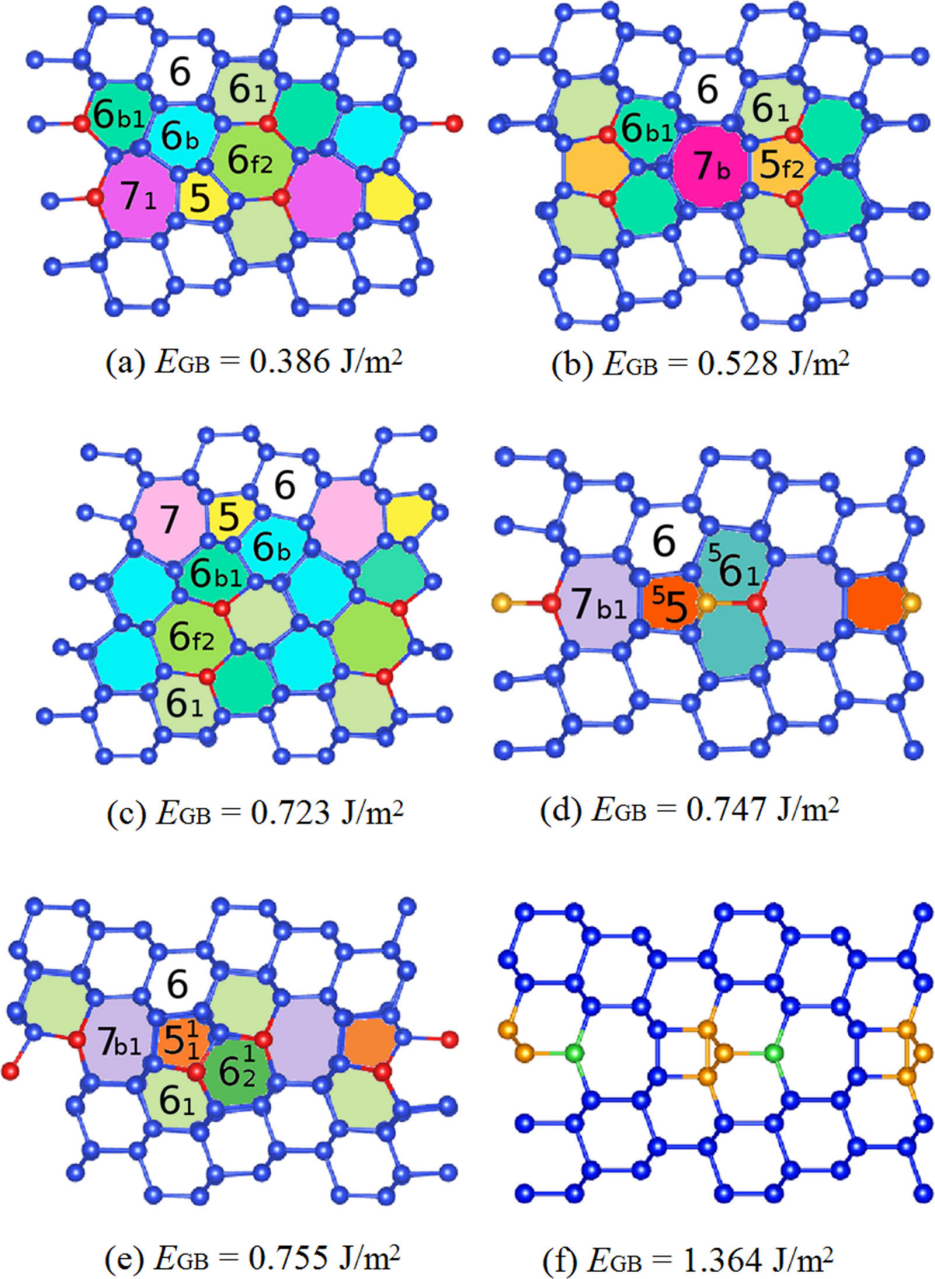
Comparison of the lowest-energy reconstructions of the Σ3(112) GB. In panels **a**–**e** we present the five lowest-energy structures. Panel **f** shows the starting structure used for the MHM simulations. For a clearer representation, we always show two unit cells along the boundary. In the figures, identical reconstructed atomic rings are filled with the same color. Red atoms form single atomic columns, orange atoms are fivefold coordinated, and green atoms have dangling bonds. We also present the GB energy and the unit atomic rings composing the interface. The same color scheme for the unit rings is used in Fig. 1.

As an example, the lowest-energy GB reconstructions of the the Σ3(112) interface, and their classification using atomic-ring units, are given in Fig. 3. The Σ3(112) interface is one of the most studied in silicon and in germanium^68,69^. The structure (d) is also known as the mirror symmetric model of Ziebarth et al.^70^, while (a) is the corresponding non-symmetric model. In agreement with ref. ^70^, we conclude that the non-symmetric model is the ground-state, with a considerably lower GB energy than its symmetric counterpart. Moreover, we find two new reconstruction patterns with intermediate interface energies, shown in panels (b) and (c). The three most stable structures exhibit two single atomic columns per unit cell (indicated by red atoms in the panels a–c of Fig. 3), while the higher-energy symmetric model has one column plus a pair of fivefold coordinated atoms (indicated by orange balls). The initial unrelaxed cell is given for comparison in panel (f): it contains fivefold coordinated atoms and dangling bonds, which are responsible for a value of the GB energy about 1 J/m^2^ larger than in the optimized ground-state structure.

In general, we find that the patterns present in the reconstructed interfaces of [110] GBs are highly dependent on the misorientation angle. In fact, for values of this angle up to 70.5°, low-energy reconstructions are composed of five-, six-, and seven-membered rings, without single atomic columns. This is confirmed by experiments, and it is reflected in experimentally based models^71,72^, originally built for diamond and then extended to other group-IV elements^73^. In contrast to these models, we do not need to introduce dangling bonds to model GBs with larger angles. Our results from structural prediction show, instead, that at larger angles the inclusion of single atomic columns is enough to restore a fourfold coordination of all silicon atoms and to reduce bond distortions. We note that, from high-resolution electron microscopy images^72^, it is often not possible to distinguish between single atomic columns and undercoordinated atoms: this fact may lead to the construction of erroneous empirical models.

Contrary to the variety of reconstructed patterns shown by the GBs with a [110] tilt axis, we find that all GBs with a [100] tilt axis are characterized by only three structural units: namely atomic spirals formed by three-, four- and five-membered rings oriented along the [100] axis. Also these results are in agreement with reported experiments^74^ and calculations^21,28,75^. We also find, for a fixed GB symmetry, that several low-energy reconstructed patterns have similar GB energies. This implies that experimental samples will exhibit a certain degree of disorder, as a mixture of low-energy patterns is expected to coexist at room temperature. Other constraints, related for example to the proximity of another GB, may lead to the formation of higher energy reconstructions.

For the majority of the GBs studied here, we find that the ground-state structure does not contain fivefold coordinated atoms. There are however exceptions to this rule, namely the Σ3(001 × 221) and the Σ19a(116) GB. Also for these anomalous cases, we can always find low-lying geometries, just slightly higher in energy, that do not present overcoordination.

As we will see in the following, the presence of fivefold coordinated atoms has important consequences on the electronic properties.

**Fig. 4:**
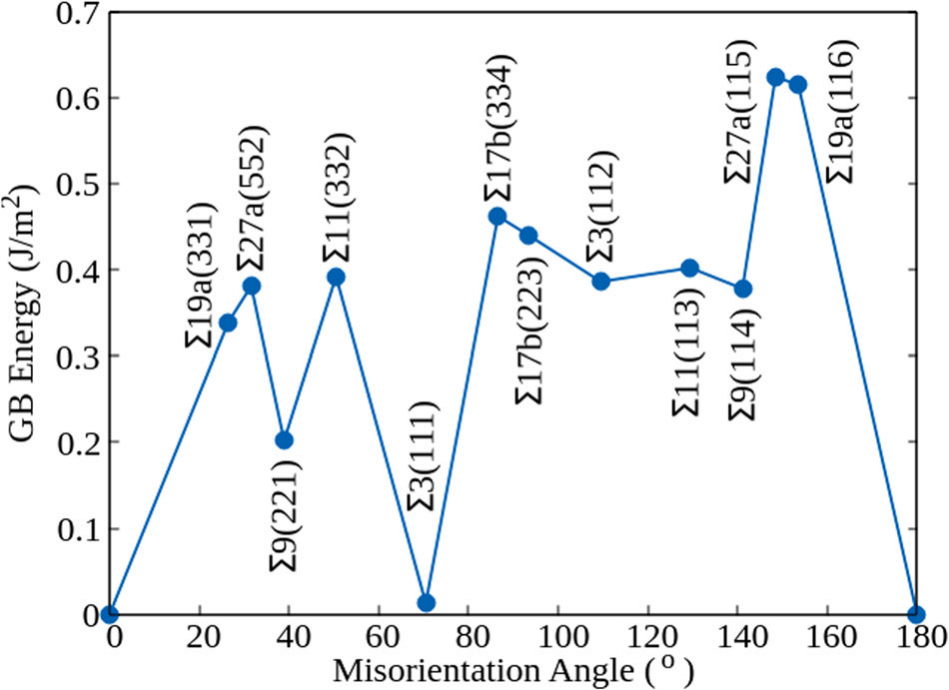
Interface energy *γ* as a function of the misorientation angle for [110] tilt GBs. Structural information on all the studied interfaces can be found in Supplementary Tables 3–5.

**Fig. 5:**
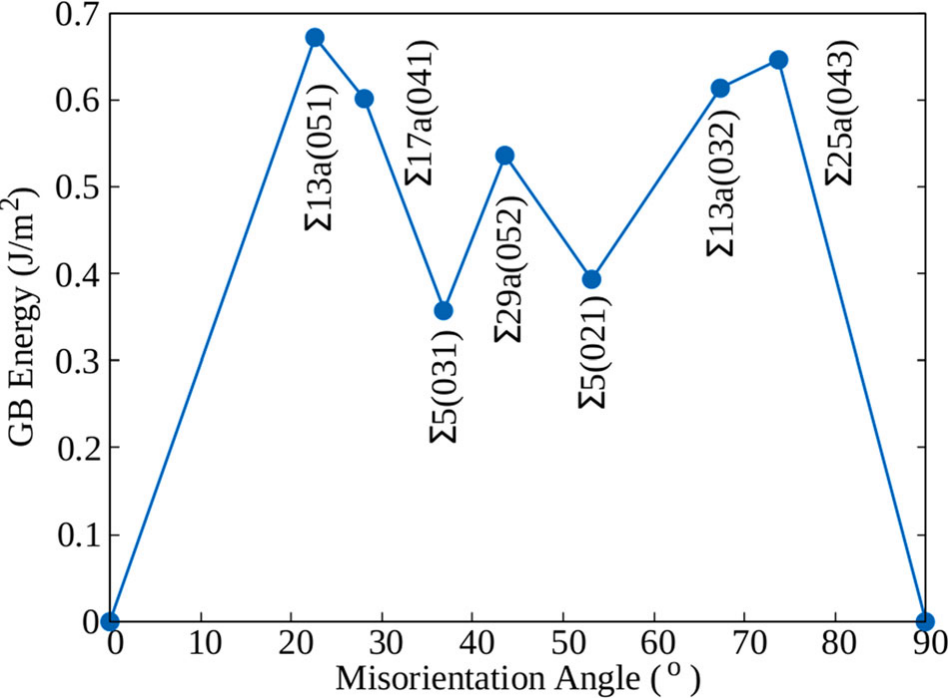
Interface energy *γ* as a function of the misorientation angle for [100] tilt GBs. Structural information on all the studied interfaces can be found in Supplementary Tables 3–5.

In Figs. 4 and 5 we plot the interface energies as a function of the misorientation angle for the ground-state reconstructed structures identified by the MHM simulations of the [110] and the [100] tilt GBs, respectively. The two asymmetric GBs Σ3(001 × 221) and Σ9(111 × 115), with interface energies of 0.497 and 0.433 J/m^2^, respectively, are not included in these graphs. The GB energies are also summarized in Table 1. We can see that the GB energies of the [110] GBs are on average lower than the GB energies of the [100] GBs. Unsurprisingly, the Σ3(111) system has by far the lowest energy^60^. In fact, this is the only system that did not present a significant surface reconstruction during the MHM simulations. The second most stable GB, according to our calculations, is the Σ9(221) interface. This can be understood by noticing that it only includes pairs of five-membered and seven-membered rings, that constitute the 5 + 7 building blocks also present in low-energy defects of carbon^65^. Interesting, the combination of 5 and 7-fold atomic rings is also the building block of M-carbon^76^ and W-carbon^77^. These patterns appear as well, for example, in low-energy reconstructions of Σ27a(552) and Σ19a(331) GBs. In contrast, the least stable GBs present more complicated arrangements of ring units, in an attempt to eliminate dangling bonds and decrease stretching and distortion of the Si–Si bonds. For example, the Σ27a(115) GB, with a GB energy of 0.623 J/m^2^, shows the pattern 5 + 5^1^ + 5_f1_ + 5_f2_ + 6 + 6_b_ + 6_1_ + 6_b1_ + 7_1_ + 7^3^ + 8_2_ + 8_b2_, while the Σ19a(116) GB, with a GB energy of 0.616 J/m^2^, has 5 + 5_f2_ + ^5^5 + 6 + 6_b_ + 6_1_ + 6_b1_ + 6_f2_ + ^5^6 + 7_1_ + 7_b1_ + ^5^7_2_ units. Drawings of all recurrent reconstruction patterns are available as Supplementary Information.

**Fig. 6:**
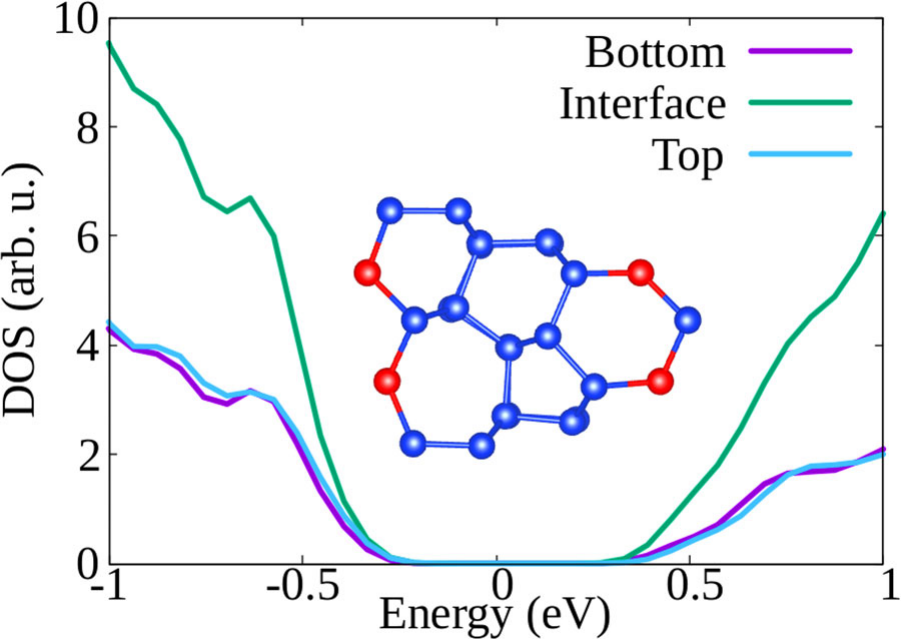
Density of states of the lowest-energy reconstruction of the Σ3(112) GB. We distinguish the contributions to the DOS coming from atoms in the top and bottom bulk layers, and atoms in the interface region. The Fermi energy is set to zero. In the inset we can see the [110] view of the reconstruction pattern. Red atoms indicate as usual single atomic columns.

**Fig. 7:**
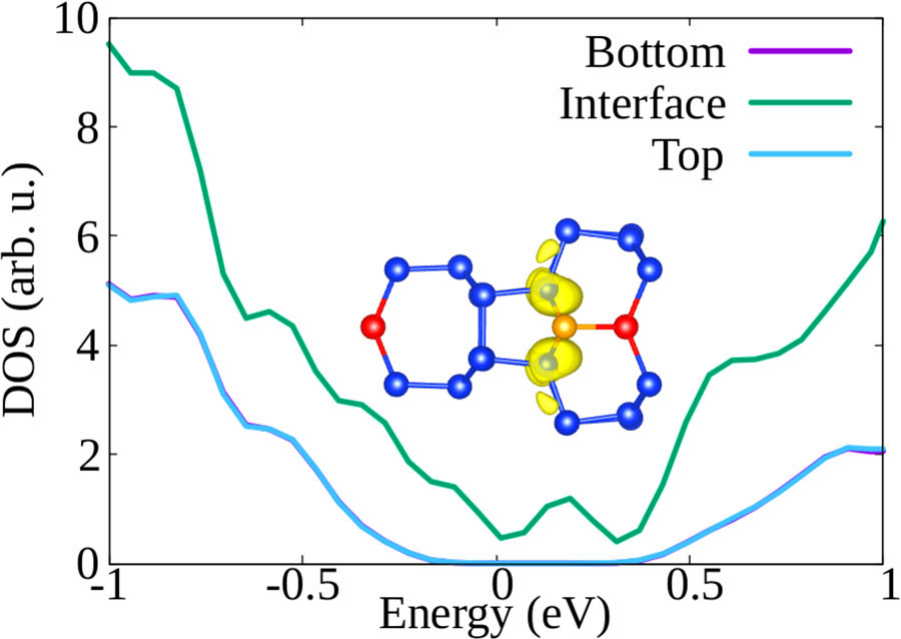
Density of states of the mirror-symmetric reconstruction of the Σ3(112) GB. We distinguish the contributions to the DOS coming from atoms in the top and bottom bulk layers, and atoms in the interface region. The Fermi energy is set to zero. In the inset we can see the [110] views of the reconstruction patterns. Red atoms indicate single atomic columns, while the orange atom is fivefold coordinated. In the inset we have also plotted the partial charge density corresponding to the DOS energy interval from −0.1 to 0.2 eV.

We now turn to the discussion of the electronic properties of the GBs and how the interface structure can affect the transport of charge carriers. We calculated the total density of states (DOS) and partial DOS, where we separate contributions from atoms belonging to bulk and interface layers, for all lowest-energy reconstructed supercells. As an example, we show in Figs. 6 and 7 the partial DOS in an energy interval around the band gap for two polymorphs of the Σ3(112) GB: the asymmetric (Fig. 6) and mirror symmetric (Fig. 7) models of Ziebarth. These structures correspond to our ground state and the fourth minimum, with GB energies of 0.386 and 0.747 J/m^2^, respectively. Both interface reconstructions have been observed in experiments^68^ and contain single atomic columns. However, the symmetric one contains fivefold coordinated atoms, that are not present in the asymmetric interface.

Looking at Fig. 6, we can observe that the contributions to the DOS coming from bulk-like atoms or interface atoms are rather similar, and similar to those of tetrahedrally coordinated atoms in monocrystalline silicon. In particular, there are no deep defect states in the gap. However, the DOS of the symmetric structure containing fivefold coordinated atoms (Fig. 7) displays a broad peak in the middle of the band-gap. In the inset of Fig. 7, where we plot the charge density coming from the electronic states around the Fermi level, we can clearly see that the electronic density corresponding to the states in the gap is localized on the fivefold coordinated atoms. We remind that localized trapping states can act as electron–hole recombination centers, and therefore are responsible for the reduction of the electrical performance of, e.g., a solar device.

Calculated DOS of lowest-energy GBs can be found in Supplementary Figs. 4 and 5. These calculations prove consistently that single atomic columns, often present at the interface of [110] GBs, and spiral rings, typical of reconstructions of [100] GBs, do not yield states in the gap, and are therefore benign for electron and hole transport in solar devices. In contrast, we found a perfect correlation between the existence of localized defect states in the gap and the presence of fivefold coordinated atoms.

In conclusion, we developed an ab initio global structural prediction method capable of building, without any experimental input, the lowest-energy reconstructions of interfaces. It is based on the minima hopping method (MHM), combined with adequate constraints to impose the presence of an interface. Energies and forces are evaluated with tight-binding parameters that can ensure, at the same time, accuracy comparable with the one of density-functional calculations and numerical efficiency. The developed approach is quite general, as it can be used to study GBs or heterogeneous interfaces of any chemical composition.

Applying this approach, we investigated a large family of tilt GBs in silicon. We found that performing a simple local relaxation of the initial geometry most often leads to very high-energy local minima. By contrast, our unbiased structural prediction approach was capable to efficiently reconstruct all interfaces, restoring fourfold coordination, with bond lengths and angles comparable to those in diamond silicon. We note that in many cases, it was essential to add or remove atoms in the interface layer to reach the ground-state structure.

The analysis of the recurrent bonding patterns in the grain boundary structures with the lowest interface energies allowed us to classify the reconstructed patterns in few families. In particular, we systematically find single atomic columns in [110] GBs and spiral rings in [100] GBs. Both these defects are electrically benign, as they do not yield states in the band-gap, which could trigger non-radiative electron–hole recombination. On the other hand, we can also find few geometries with relatively low interface energy that contain fivefold coordinated silicon atoms. These defects create localized states in the gap and are therefore expected to deteriorate charge-carrier lifetimes in electronic devices.

## Methods

**Fig. 8:**
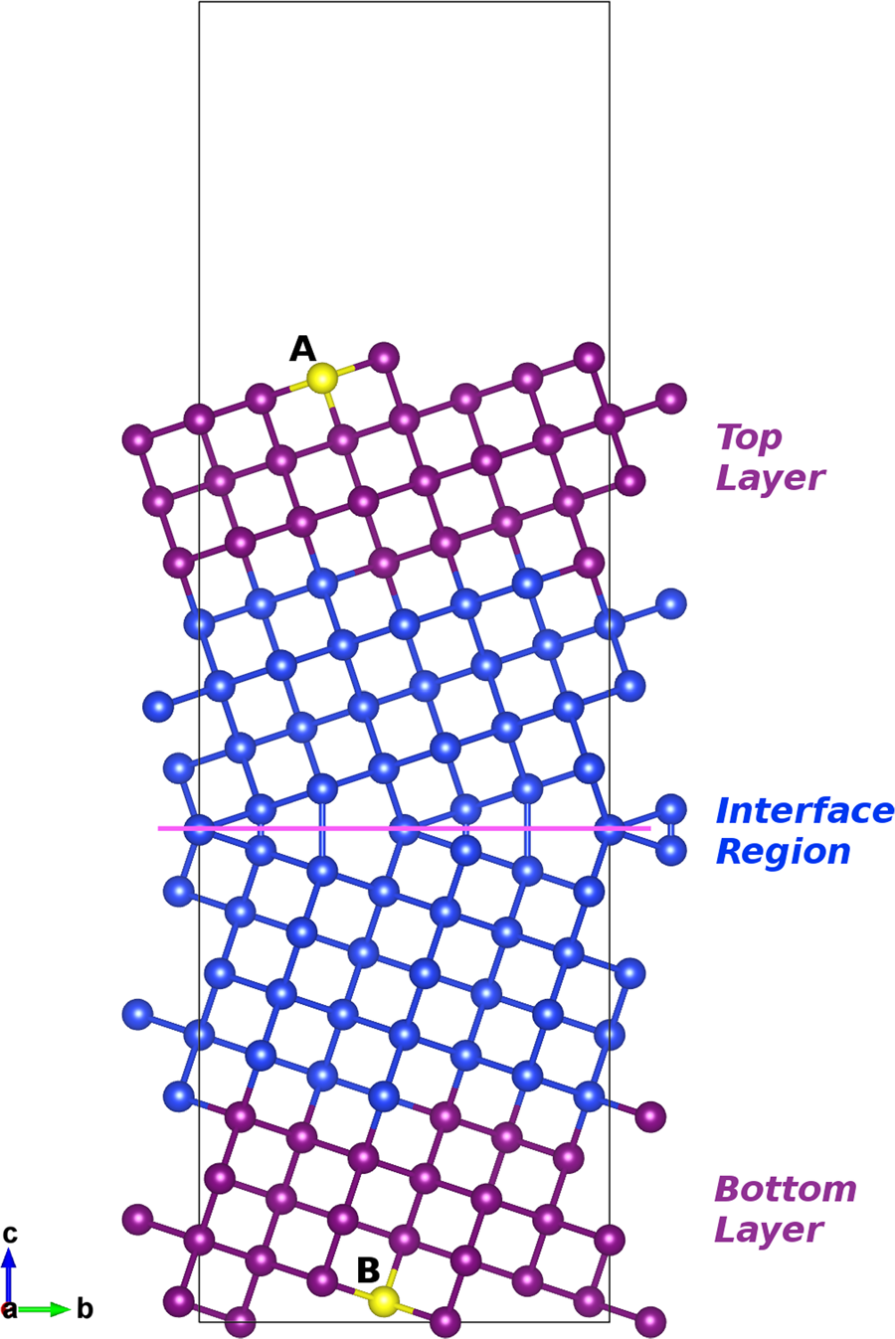
Construction of a starting supercell containing a single Σ5(021) GB. We see a cut of the supercell along the axis perpendicular to the the (021) boundary plane (pink line). The constrained layers are indicated by the magenta (and yellow) Si atoms, while blue atoms constitute the interface region and their atomic positions are optimized during the MHM simulations. Atoms A and B are highlighted in yellow as they are mentioned in the text.

All starting geometries were built using the software GB studio^78^. The coincidence site lattice model is prepared as follows: (i) Two slabs are obtained by slicing diamond silicon along one or two specific planes, indicated by their Miller indices (*hlm*), or more precisely by one of the equivalent planes of the family {*hlm*}. The thickness of the slabs must be sufficient to guarantee that a bulk-like region exists away from the surfaces. This parameter, that determines the final size of the supercell, has to be properly converged in the simulations. The two slices can be chosen to have the same orientation, and in that case only one set of Miller indices is indicated in the GB label. (ii) One of the slabs is rotated by an angle *θ* around a rotation axis [*ijk*] which lies in the cut plane (i.e., the grain is tilted). Note that choice of *θ* and of the [*ijk*] axis is constrained by the fact that we want to obtain at the end a coincidence site lattice. (iii) We fill or remove crystalline material as necessary so that the two slabs can be welded together. We also impose periodic boundary conditions, which leads to the formation of two GBs per supercell. We are interested in those cases in which we can obtain two equivalent interfaces in every supercell. (iv) The relation between the number of lattice points in the unit cell of the coincidence site lattice and the number of lattice points in a unit cell of the generating lattice determines the integer *n* that labels Σ*n*. As this number is directly connected with the rotation angle and axis, it is usually enough to specify it alone. As an illustration, we show in Fig. 8 the starting supercell of the Σ5(021) GB. In the figure, we also indicate how atoms are separated in bulk-like and interface atoms.

We implemented our constrained structural prediction in the framework of the MHM^53,54^, although it can be easily adapted to any other structural prediction algorithm. In the MHM, the energy landscape is explored using a sequence of local geometry optimization steps, followed by molecular dynamics steps in order to allow the system to escape from local minima. In the molecular dynamics steps, the direction of the initial velocities are chosen through a softening procedure. This returns the direction of a low potential barrier which, according to the Bell–Evan–Polanyi principle^79,80^, is likely to lead to a lower minimum. This new minimum can, after optimization, be accepted or not, depending on whether the energy difference to the previous minimum is below or above a certain threshold. The latter is dynamically adapted to obtain an acceptance rate of ~50%.

We explain now how the constraints are implemented, using again Fig. 8. The relative positions of each purple and yellow atoms belonging to the bulk-like layers are fixed, so that the bulk regions behave like rigid bodies. This is achieved by imposing the following conditions on the forces^81^:

1 $${{\bf{F}}}_{n}-{{\bf{F}}}_{m}=0\ ,$$

where *n* and *m* run over pairs of atoms in each bulk region. Then we choose in the top and bottom bulk layers, respectively, one atom A and one atom B. In the top layer, we imposed Eq. (1) to all pairs of atoms containing atom A, while in the bottom layer we impose the same constraint to all pairs containing atom B. We obtain therefore *N*−1 equations per layer, where *N* is the number of atoms composing each bulk layer. This kind of constraint is clearly more physical than fixing each single atomic position to the perfect crystalline bulk, as the bulk layer can move as a whole, allowing for the minimization of the strain on the atoms at the interface during their reconstruction. In the interface region, where Si atoms are colored in blue, all atomic coordinates are fully optimized.

Most often, structural prediction using the MHM is performed calculating energy and forces from first-principles using DFT. This is clearly the preferred method as DFT allows for the study of the whole periodic table with good accuracy. However, as the number of atoms in the unit cell (and, consequently, the number of local minima of the enthalpy surface) grows, DFT simulations become prohibitively long. This is certainly the case for our unit cells containing several hundred Si atoms. When good parametrizations are available, the DFT tight-binding scheme as implemented in the DFTB+ package^47^ has proved to be a valuable alternative for global structural prediction studies^55–58,65^. Tight binding combines the advantages of a fully quantum approach with a moderate computational cost. We used the tight-binding parameters developed by Huran et al.^52^ that are designed to guarantee an accurate evaluation of both energy and forces for group-IV compounds. The fitting procedure includes the generation of large unbiased training sets, and subsequent optimization of the parameters using a pattern-search method. The DFT data sets are designed to encompass a wide range of crystal structures, including characteristics of different atomic arrangements that can appear in structural prediction simulations. As target for the optimization, it is required that the formation energy and the forces on atoms calculated within tight binding reproduce the ones obtained using DFT. An extensive test of these parameters is reported in ref. ^52^.

We ran two different MHM calculations for each GB supercell, starting from different initial interface configurations. Note that the supercell lattice parameters are always kept fixed while the atomic positions are relaxed. We stopped each run after having explored more than 200 different minima. We tested that the lowest-lying minima evaluated during each run were independent of the chosen initial configuration. The most promising structures (i.e. the five lowest-energy structures of each run) were then refined at the DFT level using the code VASP^82–84^ with the Perdew–Burke–Ernzerhof (PBE) exchange-correlation functional^85^. The energy cutoff was set to 420 eV and we selected a 1 × 1 × 1 Monkhorst–Pack **k**-point^86^ grid for geometry optimization and increased it to 8 × 4 × 1 for accurate energies and densities of states. This assures that the convergence of the total energy is better than 0.01 eV per atom. We notice that the energy ordering of the different structures with the PBE is consistent with the one of tight-binding. However, it turns out that bulk silicon is overstabilized in tight-binding, leading systematically to slightly larger GB energies when compared to DFT. A comparison of DFT and tight-binding calculations is provided in Supplementary Figs. 1 and 2, as well as Supplementary Table 1. Despite the well-known limitations of the PBE functional for calculation of formation enthalpies, with errors usually at the level of ~0.2 eV/atom for dissimilar chemistries, we can rely on strong error cancellations when considering chemically similar phases^87^. In the specific case of silicon polymorphs, the reliabiliy of PBE calculations to predict phase stability has been proved by previous works^56,88^.

The quantity that the minima-hopping algorithm must optimize is not the total energy of the supercell, but the interface energy *γ*, which is defined as the difference of total energy per unit area between the supercell with a GB and a supercell with the same number of atoms of crystalline silicon. Its calculation consists in the following steps. Using periodic boundary conditions, we build a starting supercell which contains two equivalent GBs. After having calculated once for all the total energy per atom *μ*_Si_ of bulk Si, we calculate the total energy of the unrelaxed supercell ${E}_{{\rm{unrel}}}^{2{\rm{GB}}}$ containing two equivalent GBs. We then add a vacuum layer to obtain open surfaces in the normal direction, while periodic boundary conditions are maintained parallel to the GB plane. The resulting supercell has only one GB and two surfaces separated by vacuum (see Fig. 8): we calculate the energy of this unrelaxed supercell with vacuum ${E}_{{\rm{unrel}}}^{1{\rm{GB}}}$. We use now the supercell with a single GB and vacuum as a starting geometry for the MHM structural prediction, obtaining from the lowest-energy minimum ${E}_{{\rm{rel}}}^{1{\rm{GB}}}$.

The interface energy of the single unrelaxed GB is ${\gamma }^{{\rm{unrel}}}=\frac{1}{2A}\left({E}_{{\rm{unrel}}}^{2{\rm{GB}}}-N{\mu }_{{\rm{Si}}}\right)$, where *N* is the number of Si atoms in the supercell and *A* is the GB interface area. The surface energy of the unrelaxed interface with vacuum is ${\gamma }^{{\rm{surf}}}=\frac{1}{A}\left[{E}_{{\rm{unrel}}}^{1{\rm{GB}}}- N{\mu }_{{\rm{Si}}}-\frac{1}{2}\left({E}_{{\rm{unrel}}}^{2{\rm{GB}}}-N{\mu }_{{\rm{Si}}}\right)\right]$. Finally, we can evaluate the relaxed GB energy as $\gamma =\frac{1}{A}({E}_{{\rm{rel}}}^{1{\rm{GB}}}-M{\mu }_{{\rm{Si}}})-{\gamma }^{{\rm{surf}}}$, where *M* is the number of atoms in the relaxed supercell and *M* can differ from *N*, i.e. we account for the possible change of atomic density in the interface region.

We needed, of course, to build supercells of different sizes and perform consistent convergence tests to determine the ideal number of atomic layers that allows for a valid description of an isolated interface between two bulk-like crystalline domains. In Fig. 8 the thickness of the bulk layer is 14 Å while the interface is 15.4 Å thick. The vacuum layer is 10 Å thick. To perform electronic structure calculations we added hydrogen atoms to saturate the Si atoms at the surface in contact with the vacuum layer. This surface passivation, used only for calculations of the density of electronic states, prevents from having spurious surface states appearing in the band gap. The electronic DOS was also calculated with VASP, using the PBE functional^85^ and a Gaussian broadening of 0.1 eV.

## Supplementary information

Supplementary Information

## Data Availability

All data that support the findings of this study are available in the the published article and in its Supplementary Information. The crystal information files and source data for figures are available from the corresponding author upon reasonable request.

## Code availability

The code is available from the corresponding author upon reasonable request.
