# Supplementary material for: Direct insight into the structure-property relation of interfaces from constrained crystal structure prediction
Source: Nat Commun. 2021 Feb 5;12:811. doi: 10.1038/s41467-020-20855-0 (PMC7864966; doi:10.1038/s41467-020-20855-0)
Supplement: Supplementary file 1 — Supplementary Information [file 41467_2020_20855_MOESM1_ESM.pdf]

Supplementary Information for "Direct insight into the structure-property relation of interfaces from constrained crystal structure prediction"

Lin Sun,[1] Miguel A. L. Marques,[2,3] and Silvana Botti[1,3,*]

[1] *Institut für Festkörpertheorie und -optik, Friedrich-Schiller-Universität Jena, Max-Wien-Platz 1, 07743 Jena, Germany*

[2] *Institut für Physik, Martin-Luther-Universität Halle-Wittenberg, D-06099 Halle, Germany*

[3] *European Theoretical Spectroscopy Facility*

(Dated: December 11, 2020)

* silvana.botti@uni-jena.de

SUPPLEMENTARY FIGURES AND TABLES

Supplementary Note 1: Assessment of density-functional tight-binding calculations

We compare here grain boundary (GB) energies obtained with density-functional tight-binding (DFTB) using the DFTB+ package [1] and using density functional theory (DFT) as implemented in VASP [2–4]. In particular we compare the quality of the pbc-0-3 parametrization for Si distributed with the DFTB+ package and our improved parameters of Ref. 5. We selected as representative examples Σ5(031) and Σ9(221) GBs with rotation axis [100] and [110], respectively. A similar analysis is valid for the other considered GBs. Details on the calculations are given in the main manuscript.

Supplementary Table 1. GB energies in J/m^2 for Σ5(031) and Σ9(221) reconstructions, calculated with DFT and DFTB+ using two tight-binding parameter sets. In the table DFTB-param-new indicates our new parameters and DFTB-param-old indicates the pbc-0-3 parameters distributed with the DFTB+ package. We report the GB energies of the five lowest-energy structures, labeled as m1, m2, m3, m4 and m5.

	E_{GB}(J/m^2)					
	Σ5(031)			Σ9(221)		
Minima	DFTB-param-new	DFTB-param-old	DFT	DFTB-param-new	DFTB-param-old	DFT
m1	0.468	0.937	0.354	0.256	0.424	0.205
m2	0.528	1.033	0.439	0.354	0.546	0.313
m3	0.565	1.082	0.448	0.360	0.556	0.315
m4	0.586	1.092	0.502	0.399	0.600	0.359
m5	0.639	1.134	0.557	0.399	0.609	0.360

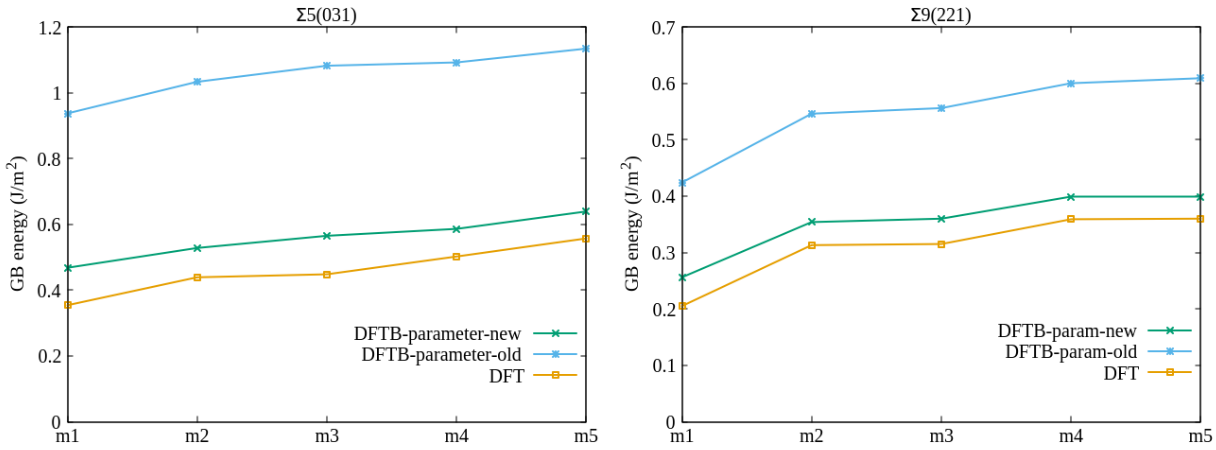

Supplementary Figure 1. GB energies of the five lowest-energy structures (m1 to m5) of Σ5(031) (left panel) and Σ9(221) (right panel), respectively. Green and blue lines are the results obtained with DFTB, using new and old sets of parameters, respectively, while the results of DFT calculations are displayed in orange.

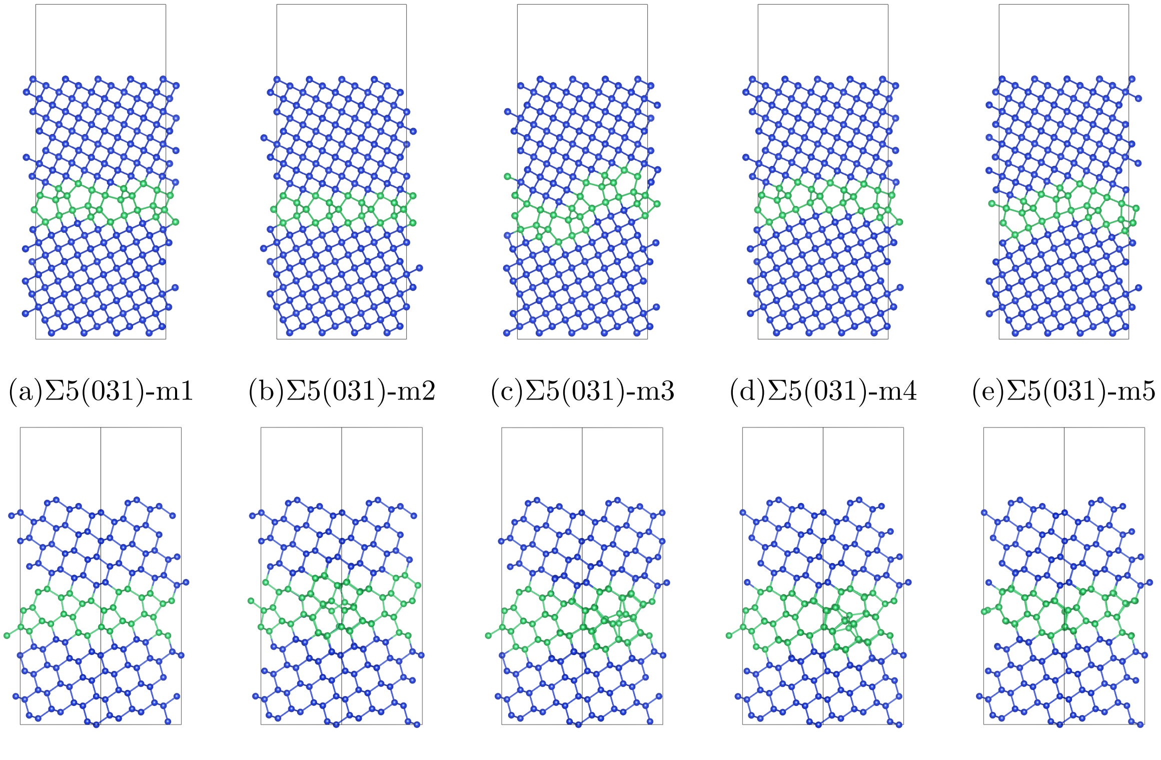

(a)Σ5(031)-m1 (b)Σ5(031)-m2 (c)Σ5(031)-m3 (d)Σ5(031)-m4 (e)Σ5(031)-m5

(f)Σ9(221)-m1 (g)Σ9(221)-m2 (h)Σ9(221)-m3 (i)Σ9(221)-m4 (j)Σ9(221)-m5

Supplementary Figure 2. The five lowest-energy structures of Σ5(031) and Σ9(221) are shown in panels (a) to (e) and (f) to (j), respectively. The label m1 to m5 indicates the energy ordering of the minima, with m1 equal to the ground state. Reconstructed bonds are highlighted in green.

Supplementary Note 2: Supercell models

We summarize in Table 2 the number of atoms belonging to the interface region, and therefore included in the minima hopping simulations, and the total number of atoms in the supercell for all considered GBs.

Supplementary Table 2. Number of atoms in the interface region N_{int} and in the whole supercell N_{total}

label	N_{int}	N_{total}
Σ13a(051)	110	204
Σ19a(331)	104	160
Σ17a(041)	184	272
Σ27a(552)	320	432
Σ5(031)	80	160
Σ9(221)	234	432
Σ29a(052)	258	464
Σ11(332)	184	352
Σ5(021)	46	94
Σ13a(032)	98	206
Σ3(111)	80	192
Σ25a(043)	294	398
Σ17b(334)	182	272
Σ17b(223)	178	266
Σ3(112)	84	188
Σ11(113)	184	352
Σ9(114)	200	288
Σ27a(115)	204	428
Σ19a(116)	208	304
Σ3(001x221)	132	284
Σ9(111x115)	224	304

Supplementary Note 3: Reconstruction patterns

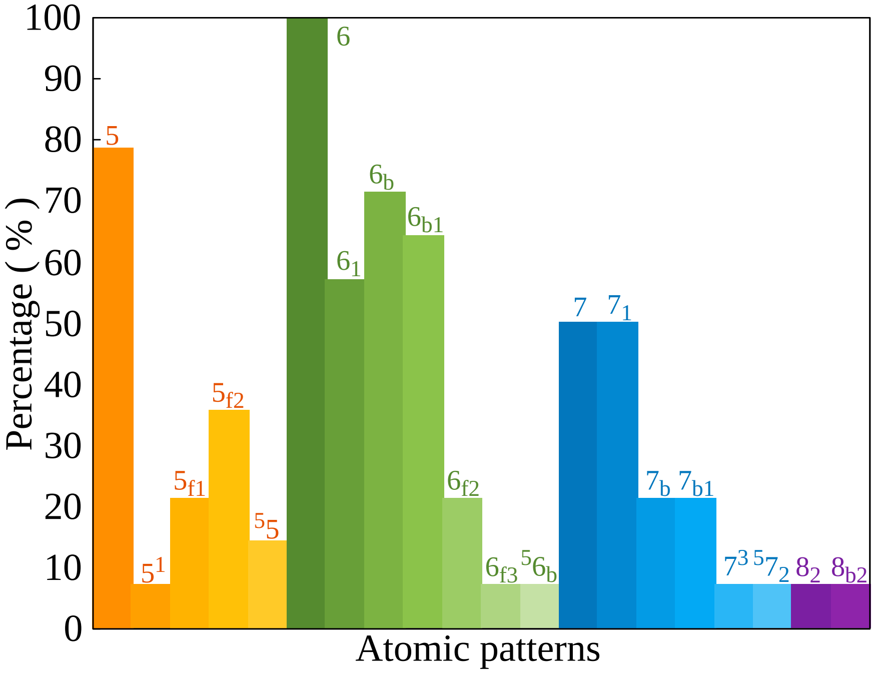

Supplementary Figure 3. Percentage of GBs that contain a specific atomic-ring unit. We consider the set of lowest-energy [110] GBs of Table 3 and Table 4. For example, all studied structures contain six-membered rings, so their occurrence is 100%, only Σ27a(115) contains 8_2 and 8_{b2} rings, and their occurrence is 7.14%.

Supplementary Table 3. Part I. Summary of misorientation angles θ, GB energies γ and structural patterns for [110] tilt GBs. Atoms forming single atomic columns are indicated in red (see the main manuscript for details).

θ	GB label	γ(J/m^2)	Patterns	
26.5°	Σ19a(331)	0.339	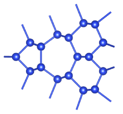	$5+6+7$
31.6°	Σ27a(552)	0.381	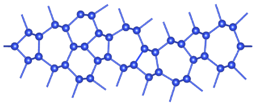	$5+6+7$
38.9°	Σ9(221)	0.202	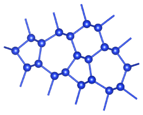	$5+6+7$
50.5°	Σ11(332)	0.392	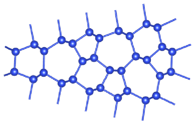	$5+6+6_b+7$
70.5°	Σ3(111)	0.013	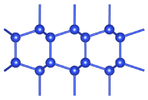	$6+6_b$
86.6°	Σ17b(334)	0.462	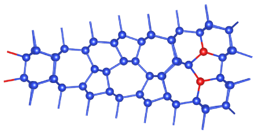	$5+5_{f2}$<br>$6+6_b+6_1+6_{b1}$<br>$7+7_b+7_1$
93.4°	Σ17b(223)	0.449	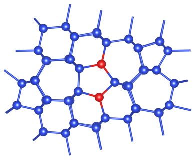	$5+5_{f2}$<br>$6+6_b+6_1+6_{b1}$<br>$7+7_b+7_1$
109.5°	Σ3(112)	0.386	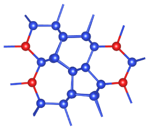	5<br>$6+6_b+6_{b1}+6_{f2}$<br>7_1

Supplementary Table 4. Part II. Summary of misorientation angles θ, GB energies γ and structural patterns for [110] tilt GBs. Atoms forming single atomic columns are indicated in red, orange atoms are fivefold coordinated (see the main manuscript for details).

θ	GB label	$E_{\mathrm{GB}}(\mathrm{J/m^2})$		patterns
129.5°	Σ11(113)	0.402	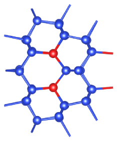	$6 + 6_1 + 6_{b1} + 6_{f2}$
141.1°	Σ9(114)	0.378	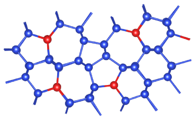	$5 + 5_{f1}$<br>$6 + 6_b + 6_1 + 6_{b1}$<br>$7_1 + 7_{b1}$
148.4°	Σ27a(115)	0.623	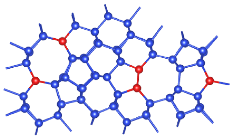	$5 + 5^1 + 5_{f1} + 5_{f2}$<br>$6 + 6_b + 6_1 + 6_{b1}$<br>$7_1 + 7^3$<br>$8_2 + 8_{b2}$
153.5°	Σ19a(116)	0.616	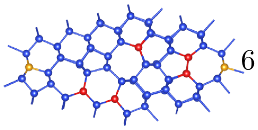	$5 + 5_{f2} + {}^5 5$<br>$6 + 6_b + 6_1 + 6_{b1} + 6_{f2} + {}^5 6$<br>$7_1 + 7_{b1} + {}^5 7_2$
70.5°	Σ3(001x221)	0.497	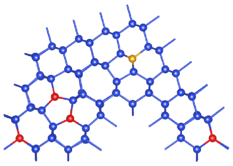	$5_{f2} + {}^5 5$<br>$6 + 6_b + 6_1 + 6_{b1} + {}^5 6_b$<br>$7_b + 7_{b1}$
38.9°	Σ9(111x115)	0.433	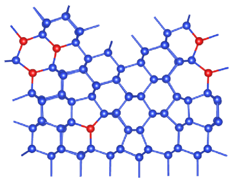	$5 + 5_{f1}$<br>$6 + 6_b + 6_1 + 6_{b1} + 6_{f3}$<br>$7 + 7_1$

Supplementary Table 5. Summary of misorientation angles θ, GB energies γ and structural patterns for [100] tilt GBs.

θ	GB label	$E_{\mathrm{GB}}(\mathrm{J/m^2})$	patterns
22.6°	Σ13a(051)	0.672	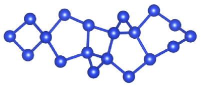
28.1°	Σ17a(041)	0.601	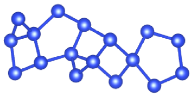
36.9°	Σ5(031)	0.358	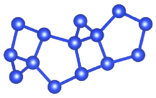
43.6°	Σ29a(052)	0.536	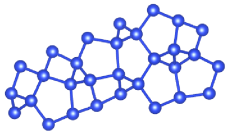
53.1°	Σ5(021)	0.393	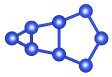
67.4°	Σ13a(032)	0.614	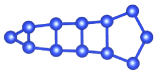
73.7°	Σ25a(043)	0.646	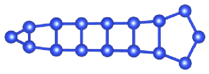

Supplementary Note 4: Density of electronic states

We show in the following figures calculated density of states close to the band edges for the lowest energy GB reconstructions. We indicate in red if the structure presents fivefold coordinated atoms at the interface.

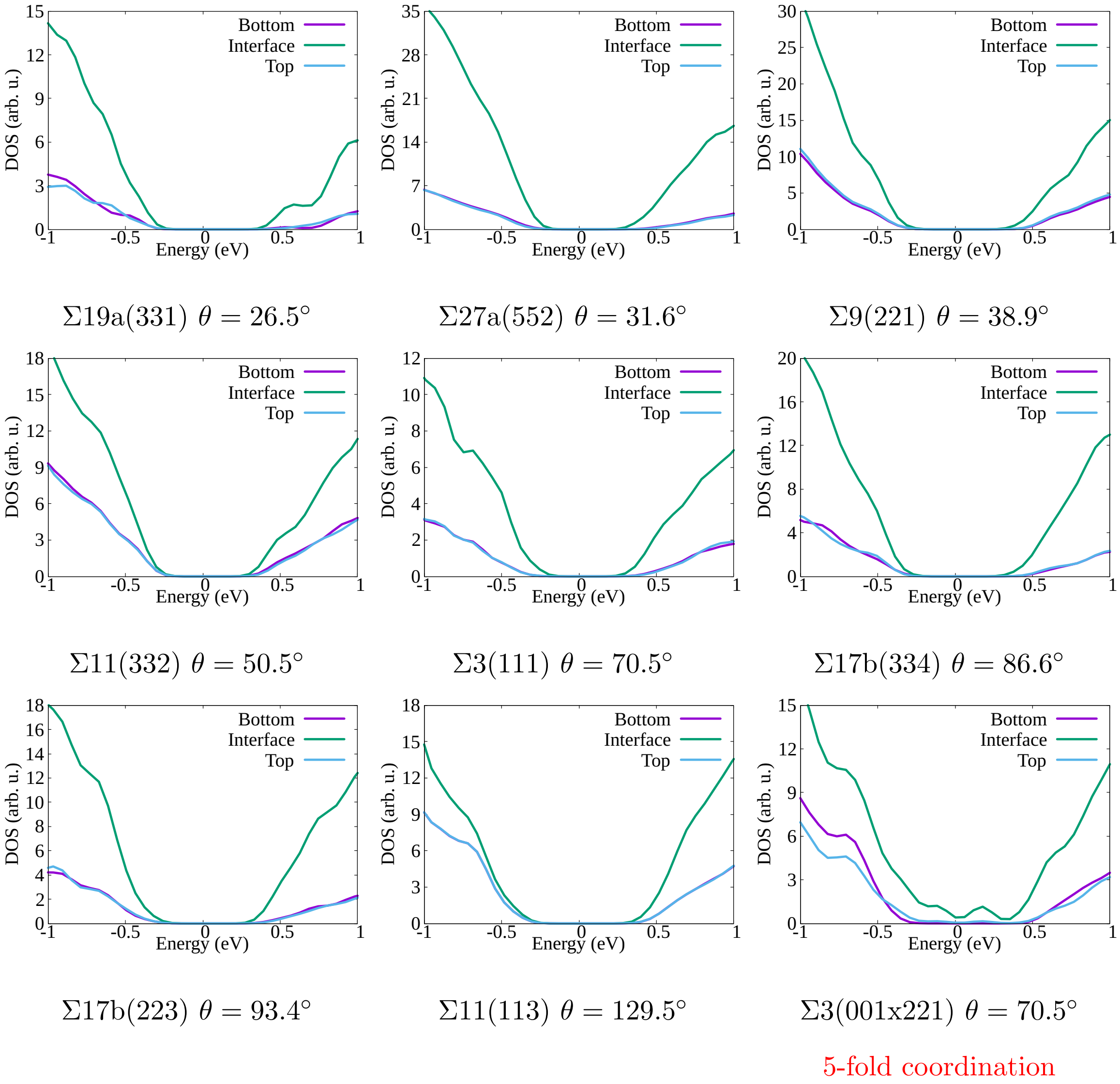

Supplementary Figure 4. Part I. Densities of states close to the band gap. The Fermi energy is set to zero.

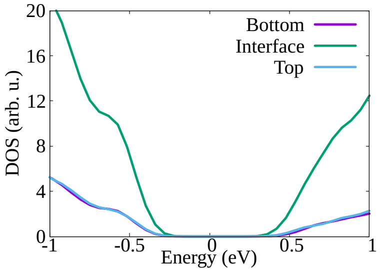

$\Sigma9(114)\ \theta = 141.1°$

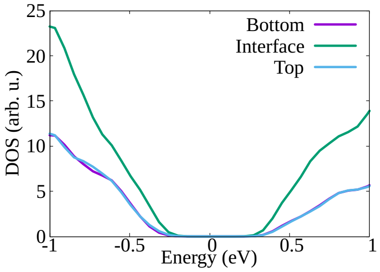

$\Sigma27a(115)\ \theta = 148.4°$

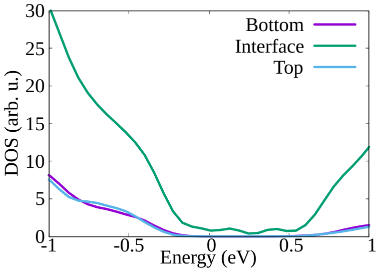

$\Sigma19a(116)\ \theta = 153.5°$

5-fold coordination

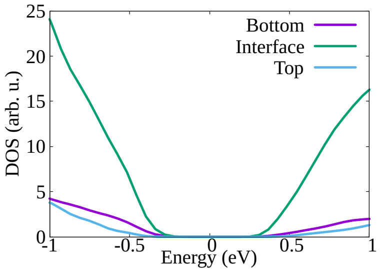

$\Sigma9(111x115)\ \theta = 38.9°$

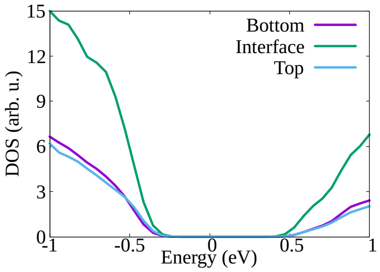

$\Sigma13a(051)\ \theta = 22.62°$

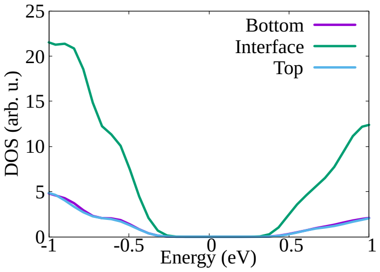

$\Sigma17a(041)\ \theta = 28.07°$

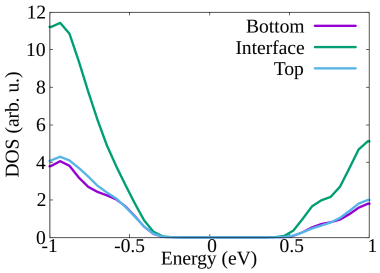

$\Sigma5(031)\ \theta = 36.86°$

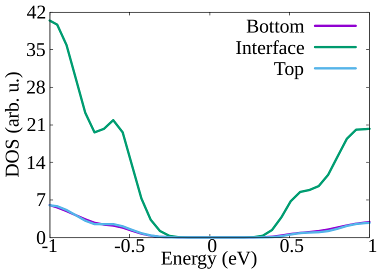

$\Sigma29a(052)\ \theta = 43.60°$

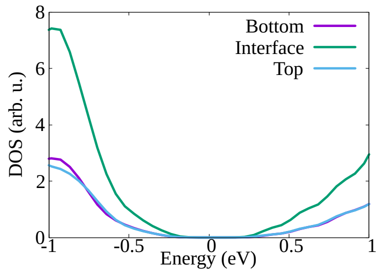

$\Sigma5(021)\ \theta = 53.14°$

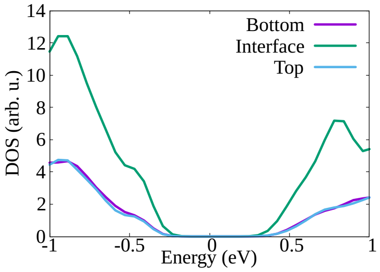

$\Sigma13a(032)\ \theta = 67.38°$

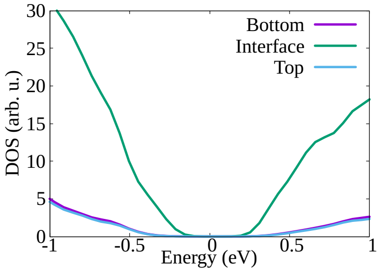

$\Sigma25a(043)\ \theta = 73.74°$

Supplementary Figure 5. Part II. Densities of states close to the band gap. The Fermi energy is set to zero.

SUPPLEMENTARY REFERENCES

---

[1] Aradi, B., Hourahine, B. & Frauenheim, T. DFTB+, a sparse matrix-based implementation of the DFTB method. *J. Phys. Chem. A* **111**, 5678–5684 (2007). URL `https://doi.org/10.1021/jp070186p`.

[2] Kresse, G. & Hafner, J. Ab initio molecular dynamics for liquid metals. *Phys. Rev. B* **47**, 558–561 (1993). URL `https://link.aps.org/doi/10.1103/PhysRevB.47.558`.

[3] Kresse, G. & Furthmüller, J. Efficient iterative schemes for ab initio total-energy calculations using a plane-wave basis set. *Phys. Rev. B* **54**, 11169 (1996). URL `https://link.aps.org/doi/10.1103/PhysRevB.54.11169`.

[4] Kresse, G. & Joubert, D. From ultrasoft pseudopotentials to the projector augmented-wave method. *Phys. Rev. B* **59**, 1758 (1999). URL `https://link.aps.org/doi/10.1103/PhysRevB.59.1758`.

[5] Huran, A. W., Steigemann, C., Frauenheim, T., Aradi, B. & Marques, M. A. L. Efficient automatized density-functional tight-binding parametrizations: Application to group IV elements. *J. Chem. Theory Comput.* **14**, 2947–2954 (2018). URL `https://doi.org/10.1021/acs.jctc.7b01269`.
